# Supplementary material for: Antidepressant prescribing inequalities in people with comorbid depression and type 2 diabetes: A UK primary care electronic health record study
Source: PLoS One. 2024 Nov 5;19(11):e0309153. doi: 10.1371/journal.pone.0309153 (PMC11537397; doi:10.1371/journal.pone.0309153)
Supplement: Attachment — Submitted filename: DK review - PONE-D-24-10322.pdf [file pone.0309153.s001.pdf]

PLOS ONE

Antidepressant prescribing inequalities in people with comorbid depression and type 2 diabetes: a UK primary care electronic health record study

--Manuscript Draft--

Manuscript Number:	PONE-D-24-10322
Article Type:	Research Article
Full Title:	Antidepressant prescribing inequalities in people with comorbid depression and type 2 diabetes: a UK primary care electronic health record study
Short Title:	TITLE: Antidepressant prescribing inequalities in people with comorbid depression and type 2 diabetes
Corresponding Author:	Annie Jeffery<br>University College London<br>London, UNITED KINGDOM
Keywords:	Type 2 diabetes; Depression; antidepressant; inequality; primary care; multimorbidity
Abstract:	Aims: To compare the likelihood of being prescribed an antidepressant in depressed individuals with and without type 2 diabetes.<br>Methods: We performed a matched cohort study using primary care record data from the UK Clinical Practice Research Datalink. We used multivariable logistic regression to compare antidepressant prescribing during the first five years of starting oral antidiabetic medication to a comparison group without type 2 diabetes, matched based on GP practice, age and sex. We performed subgroup analyses stratified by sex, age and ethnicity.<br>Results: People with type 2 diabetes and depression were 75% less likely to be prescribed an antidepressant compared to people with depression alone (odds ratio (OR) 0.25, 95% confidence interval (CI) 0.25 to 0.26). This difference was greater in males (OR 0.23, 95% CI, 0.22 to 0.24), people older than 56 years (OR 0.23, 95% CI, 0.22 to 0.24), or from a minoritised ethnic background (Asian OR 0.14, 95% CI 0.12-0.14; Black OR 0.12, 95% CI 0.09-0.14).<br>Conclusions: There may be inequalities in access to antidepressant treatment for people with type 2 diabetes, particularly those who are male, older or from minoritised ethnic backgrounds.
Order of Authors:	Yutung Ng
	Joseph Hayes
	Annie Jeffery
Additional Information:	
**Question**	**Response**
**Financial Disclosure**<br><br>Enter a financial disclosure statement that describes the sources of funding for the work included in this submission. Review the submission guidelines for detailed requirements. View published research articles from *PLOS ONE* for specific examples.<br><br>This statement is required for submission and **will appear in the published article** if the submission is accepted. Please make	The author(s) received no specific funding for this work.

Powered by Editorial Manager® and ProduXion Manager® from Aries Systems Corporation

sure it is accurate.<br><br>**Funded studies**<br>Enter a statement with the following details:<br>• Initials of the authors who received each award<br>• Grant numbers awarded to each author<br>• The full name of each funder<br>• URL of each funder website<br>• Did the sponsors or funders play any role in the study design, data collection and analysis, decision to publish, or preparation of the manuscript?<br><br>Did you receive funding for this work?	
**Competing Interests**<br><br>Use the instructions below to enter a competing interest statement for this submission. On behalf of all authors, disclose any competing interests that could be perceived to bias this work—acknowledging all financial support and any other relevant financial or non-financial competing interests.<br><br>This statement is required for submission and **will appear in the published article** if the submission is accepted. Please make sure it is accurate and that any funding sources listed in your Funding Information later in the submission form are also declared in your Financial Disclosure statement.<br><br>View published research articles from *PLOS ONE* for specific examples.	The authors have declared that no competing interests exist.

**NO authors have competing interests**<br><br>Enter: *The authors have declared that no competing interests exist.*<br><br>**Authors with competing interests**<br><br>Enter competing interest details beginning with this statement:<br><br>*I have read the journal's policy and the authors of this manuscript have the following competing interests: [insert competing interests here]*<br><br>* typeset	
**Ethics Statement**<br><br>Enter an ethics statement for this submission. This statement is required if the study involved:<br><br>• Human participants<br>• Human specimens or tissue<br>• Vertebrate animals or cephalopods<br>• Vertebrate embryos or tissues<br>• Field research<br><br>Write "N/A" if the submission does not require an ethics statement.<br><br>General guidance is provided below. Consult the submission guidelines for detailed instructions. **Make sure that all information entered here is included in the Methods section of the manuscript.**	N/A

**Format for specific study types**<br><br>**Human Subject Research (involving human participants and/or tissue)**<br>• Give the name of the institutional review board or ethics committee that approved the study<br>• Include the approval number and/or a statement indicating approval of this research<br>• Indicate the form of consent obtained (written/oral) or the reason that consent was not obtained (e.g. the data were analyzed anonymously)<br><br>**Animal Research (involving vertebrate animals, embryos or tissues)**<br>• Provide the name of the Institutional Animal Care and Use Committee (IACUC) or other relevant ethics board that reviewed the study protocol, and indicate whether they approved this research or granted a formal waiver of ethical approval<br>• Include an approval number if one was obtained<br>• If the study involved *non-human primates*, add *additional details* about animal welfare and steps taken to ameliorate suffering<br>• If anesthesia, euthanasia, or any kind of animal sacrifice is part of the study, include briefly which substances and/or methods were applied<br><br>**Field Research**<br><br>Include the following details if this study involves the collection of plant, animal, or other materials from a natural setting:<br>• Field permit number<br>• Name of the institution or relevant body that granted permission	
**Data Availability**<br><br>Authors are required to make all data underlying the findings described fully available, without restriction, and from the time of publication. PLOS allows rare exceptions to address legal and ethical concerns. See the PLOS Data Policy and FAQ for detailed information.	No - some restrictions will apply

A Data Availability Statement describing where the data can be found is required at submission. Your answers to this question constitute the Data Availability Statement and **will be published in the article**, if accepted.<br><br>**Important:** Stating 'data available on request from the author' is not sufficient. If your data are only available upon request, select 'No' for the first question and explain your exceptional situation in the text box.<br><br>Do the authors confirm that all data underlying the findings described in their manuscript are fully available without restriction?	
**Describe where the data may be found in full sentences. If you are copying our sample text, replace any instances of XXX with the appropriate details.**<br><br>• If the data are **held or will be held in a public repository**, include URLs, accession numbers or DOIs. If this information will only be available after acceptance, indicate this by ticking the box below. For example: *All XXX files are available from the XXX database (accession number(s) XXX, XXX.*).<br>• If the data are all contained **within the manuscript and/or Supporting Information files**, enter the following: *All relevant data are within the manuscript and its Supporting Information files.*<br>• If neither of these applies but you are able to provide **details of access elsewhere**, with or without limitations, please do so. For example:<br><br>*Data cannot be shared publicly because of [XXX]. Data are available from the XXX Institutional Data Access / Ethics Committee (contact via XXX) for researchers who meet the criteria for access to confidential data.*<br><br>*The data underlying the results presented in the study are available from (include the name of the third party*	Data cannot be shared publicly because it is person-level electronic health record data. Data are available upon application from the CPRD - www.cprd.com for researchers who meet the criteria for access to confidential data.

Powered by Editorial Manager® and ProduXion Manager® from Aries Systems Corporation

and contact information or URL).<br>• This text is appropriate if the data are owned by a third party and authors do not have permission to share the data.<br><br>* typeset	
Additional data availability information:	

Powered by Editorial Manager® and ProduXion Manager® from Aries Systems Corporation

Manuscript

Click here to access/download;Manuscript;final version AD prescribing in T2DM FINAL plos one.docx

TITLE: Antidepressant prescribing inequalities in people with comorbid depression and type 2 diabetes: a UK primary care electronic health record study

Authors: Ng Y[1] MSc, Hayes J[1] PhD, Jeffery A[1] PhD.

1. Division of Psychiatry, University College London, Gower St, London WC1E 6BT, UK.

Corresponding author: Annie Jeffery, annie.jeffery@ucl.ac.uk;

ORCID ID: 0000-0001-6218-0711

ABSTRACT

Aims: To compare the likelihood of being prescribed an antidepressant in depressed individuals with and without type 2 diabetes.

Methods: We performed a matched cohort study using primary care record data from the UK Clinical Practice Research Datalink. We used multivariable logistic regression to compare antidepressant prescribing during the first five years of starting oral antidiabetic medication to a comparison group without type 2 diabetes, matched based on GP practice, age and sex. We performed subgroup analyses stratified by sex, age and ethnicity.

Results: People with type 2 diabetes and depression were 75% less likely to be prescribed an antidepressant compared to people with depression alone (odds ratio (OR) 0.25, 95% confidence interval (CI) 0.25 to 0.26). This difference was greater in males (OR 0.23, 95% CI, 0.22 to 0.24), people older than 56 years (OR 0.23, 95% CI, 0.22 to 0.24), or from a minoritised ethnic background (Asian OR 0.14, 95% CI 0.12-0.14; Black OR 0.12, 95% CI 0.09-0.14).

Conclusions: There may be inequalities in access to antidepressant treatment for people with type 2 diabetes, particularly those who are male, older or from minoritised ethnic backgrounds.

Key words: type 2 diabetes, depression, antidepressant, inequality, primary care, multimorbidity

INTRODUCTION

People with type 2 diabetes are approximately three times more likely to suffer from depression than people without type 2 diabetes (1). When the conditions are comorbid, depression has been shown to be associated with poor glycemic control (2), the development of serious diabetic complications (3) and premature mortality (4). Thus, the successful treatment of depression is important to both physical and mental health.

Antidepressants are recommended in UK guidelines to treat moderate to severe depression (5). For people who have physical comorbidities, guidelines are vague, and not condition-specific – advising the clinician be ware of possible drug-drug or drug-disease interactions (6). In people with type 2 diabetes, antidepressants have been found in a Cochrane review to both reduce depressive symptoms and improve glycaemic control (7). However, antidepressants have common side effects that may be of particular concern for people with type 2 diabetes; such as increased weight, cardiac symptoms, nausea and drug-drug interactions (8). There is also very limited evidence concerning the effects of antidepressants on long-term physical health in people with type 2 diabetes (9). This can make for difficult prescribing decisions, which could result in inappropriate prescribing or, alternatively, inequalities in access to treatment.

A retrospective cohort study using primary care data in the UK found that people with type 2 diabetes were more likely to be prescribed an antidepressant than people without type 2 diabetes (10). However, this study did not measure whether or not patients had depression. Therefore, the higher rates of antidepressant prescribing are likely to reflect the higher prevalence of depression in people with type 2 diabetes. There is no other research in the UK, to the best of our knowledge, that investigates the difference in antidepressant prescribing in individuals with depression, between people with and without type 2 diabetes.

There is evidence in the general population that people with certain demographic characteristics are more likely to be prescribed antidepressants (female (11,12); older age (12); and White ethnicity (13)). These inequalities could be of particular relevance to people with type 2 diabetes, which is more likely to affect males (14) and people from certain minoritised ethnic backgrounds (15). It could also be more problematic in older adults who are more likely to have additional comorbidities (16). However, none of the abovementioned demographic factors have been examined relative to antidepressant prescribing in individuals with and without type 2 diabetes.

We aimed to address this gap in the evidence using UK primary care data to compare, in people with depression, the likelihood of being prescribed an antidepressant in people with comorbid type 2 diabetes, and people without type 2 diabetes. We hypothesized that people with comorbid depression and type 2 diabetes would be more likely to be prescribed antidepressants than those without type 2 diabetes, due to the increased need to successfully treat depression in this patient group.

Our secondary aim was to investigate the sociodemographic factors that could influence the relationship between type 2 diabetes and antidepressant prescribing – in particular, sex, age, and ethnicity. We hypothesised that the increased prevalence of antidepressant prescribing in people with type 2 diabetes, would be greatest for individuals who were female, older and of White ethnicity.

METHODS

Study design

We used a matched cohort study design to investigate the likelihood of individuals with depression being prescribed an antidepressant, in those with type 2 diabetes, compared to those without type 2 diabetes.

Data source

We used primary care data from the UK Clinical Practice Research Datalink (CPRD) (17). This data set encompasses anonymous electronical health records (EHRs) of approximately 60 million patients (16 million current patients) across the UK. The CPRD combines two databases, CPRD Gold and CPRD Aurum, which contain records from different EHR software (Vision and EMIS, respectively). We combined both CPRD Gold and Aurum in our analysis. The CPRD has been shown to be representative of the UK population with regards to sex, age and ethnicity (18,19).

Data for this project were accessed from 1 January to 1 September 2022. Access to the data was approved by the Independent Scientific Advisory Committee of CPRD (protocol no. 21_001648). All data sent to the CPRD is anonymised.

Study population

All participants in our study were registered with a CPRD GP practice for at least one year between the years 2000 to 2018 and had a record of depression (diagnosis, symptom or process of care – such as "depression monitoring letter sent") during the EHR follow-up.

Exposure and comparison

The exposed group comprised individuals with depression who also had type 2 diabetes. We identified people with type 2 diabetes as those starting oral antidiabetic medication for the first time, as this was a standardised point in time to start follow-up. We required a 6-month period after their date of registration at a CPRD primary care practice without any oral antidiabetic prescriptions, before the date of their first oral antidiabetic prescription to indicate incident prescribing. We also required our exposed group to have at least 2 diagnostic tests for type 2 diabetes above the threshold used to indicate type 2 diabetes, as some antidiabetic medications (namely metformin) may be used to treat other conditions. We excluded people who were likely to have type 1 diabetes, by excluding those with an insulin

prescription less than 6 months before the first oral antidiabetic prescription. We excluded people with probable gestational diabetes only, by excluding women who only had oral antidiabetic medication prescribed during periods of pregnancy. We set the index date of the exposed group as the date of their first oral antidiabetic prescription.

We included in our comparison group individuals with depression, but no record related to diabetes or any antidiabetic medication prescribed. We matched up to four individuals without type 2 diabetes randomly to people with type 2 diabetes, based on whether they were registered at the same GP practice, age (rolling 5 years) and sex. We set the index date of the comparison group as the date of their match's first oral antidiabetic prescription.

In both the exposed and comparison groups, we excluded individuals prescribed antidepressants prior to the index date.

We followed up all individuals in the study for 5 years, and censored anyone during this time who died or de-registered from their GP practice. We required that both the exposed and comparison groups had a record of depression during the 5 - year follow-up period.

Outcome

Our outcome was the receipt of one or more antidepressant prescription(s) recorded during the follow-up period. We considered the following antidepressant medications: selective serotonin re-uptake inhibitors (citalopram, escitalopram, fluoxetine, fluvoxamine, paroxetine, sertraline), serotonin-noradrenaline reuptake inhibitors (duloxetine, venlafaxine), tricyclic antidepressants (amitriptyline >= 50mg, amoxapine, clomipramine, dosulepin, imipramine, lofepramine, maprotiline, nortriptyline >= 50mg, trimipramine), monoamine oxidase inhibitors (isocarboxazid, moclobemide, phenelzine, tranylcypromine) or other antidepressant agents (agomelatine, mianserin, mirtazapine, nefazodone, oxitriptan, reboxetine, trazodone, tryptophan).

Confounders

We considered the following variables as confounders: sex (male or female), age (in years), ethnicity (categorised as Asian, Black, Mixed, Other, White, and Missing), and GP practice.

Statistical analysis

We used logistic regression to model the association between type 2 diabetes and being prescribed antidepressant medication within 5 years of the study index date. We first performed a univariable analysis and then added all aforementioned confounders in a multivariable model. Clustering by GP practice was accounted for by including GP practice ID as a strata term in multilevel models. We also performed interaction tests between the main exposure of type 2 diabetes, and age, sex and ethnicity. If

we found any evidence of interactions (p-value <0.05), we performed subgroup analyses, stratifying by the aforementioned categories (for sex and ethnicity) and above/below the median age

RESULTS

We included a total of 113,812 individuals with depression. Of these, 23,171 (20.3%) had type 2 diabetes and 90,641 (79.6%) did not have type 2 diabetes. Table 1 provides the details of the baseline characteristics of the patients included the analysis, stratified by whether or not they had type 2 diabetes. Overall, 48.4% of patients included were female, the median age was 56 years (interquartile range, 46–66), and the majority (53.1%) were of White ethnicity. Patients with type 2 diabetes were more likely to be of Asian (8.7% vs 4.6%) or Black (3.3% vs 1.9%) ethnicity.

The results for our comparison of antidepressant prescribing according to whether or not an individual had type 2 diabetes are presented in Table 2. After adjusting for confounders, individuals with comorbid depression and type 2 diabetes were 75% less likely to be prescribed an antidepressant than those who had depression without type 2 diabetes (odds ratio (OR) 0.25, 95% CI, 0.25 to 0.26). There was no evidence of a change between the univariable and multivariable models.

We found evidence of an interaction between sex and type 2 diabetes, with regards to being prescribed an antidepressant ($p < 0.0001$). Therefore, we stratified our multivariable analysis by sex (Table 3). Being male further decreased the likelihood of being prescribed an antidepressant if the individual had type 2 diabetes, compared to if they did not have type 2 diabetes (77% less likely in men – OR 0.23, 95% CI, 0.22 to 0.24; compared to 72% less likely in women – OR 0.28, 95% CI, 0.27 to 0.29).

We found an interaction between age and type 2 diabetes, regarding antidepressants prescription ($p < 0.0001$). Therefore, we stratified our primary multivariable analysis by age (Table 3). Being over the age of 56 years further decreased the likelihood of being prescribed an antidepressant if the individual type 2 diabetes, compared to if they did not have type 2 diabetes (77% less likely in people aged over 56 years – OR 0.23, 95% CI, 0.22 to 0.24; compared to 72% less likely in people aged under 56 years – OR 0.28, 95% CI, 0.27 to 0.29).

We found an interaction between ethnicity and type 2 diabetes, with regards to being prescribed an antidepressant ($p < 0.0001$). Therefore, we stratified our primary multivariable analysis by ethnicity (Table 3). Being from an ethnic minority further decreased the likelihood of being prescribed an antidepressant if the individual had type 2 diabetes, compared to if they did not have type 2 diabetes (70% lower in the majority of people with White ethnicity – OR 0.30, 95% CI, 0.29 to 0.32; compared to 86% lower in people of Asian ethnicity – OR 0.14, 95% CI, 0.12 to 0.16; 85% lower in people of Black ethnicity – OR 0.15, 95% CI, 0.09 to 0.24; 85% lower in people of Mixed ethnicity – OR 0.15,

95% CI 0.09 to 0.24; 85% lower in people of Other ethnicity – OR 0.15, 95% CI 0.10 to 0.22; and 76% lower in people with missing ethnicity).

DISCUSSION

Summary

Our study was the first to investigate the difference in antidepressant prescribing in individuals with depression between those who did or did not have comorbid type 2 diabetes. We had hypothesised that people with type 2 diabetes would be more likely to be prescribed antidepressants, however, we found the opposite. People with comorbid depression and type 2 diabetes were 74% less likely to be prescribed antidepressants than people who had depression without type 2 diabetes, after adjusting for age, sex, ethnicity and GP practice. We found that the reduced likelihood of prescribing in people with type 2 diabetes was even further reduced in people who were male, older and from an ethnic minority background.

Strengths and limitations

We used data from a large primary care database that is representative of the UK general population. This allowed us to investigate antidepressant prescribing as it happens in the real world, with a large enough sample size to investigate demographic interactions with type 2 diabetes and antidepressant prescribing. Unlike other previous studies (10), all participants in our study had a record of depression. This means that it is not differences in depression rates driving our findings, but is truly difference in antidepressants prescribing for depression between individuals with type 2 diabetes and those without.

We are confident in the validity of our antidepressant prescribing outcome, as antidepressant prescribing in the UK is through primary care, and all prescriptions are issued electronically and therefore automatically recorded on a patient's record. However, we were unable to see reasons for the decision to prescribe an antidepressant (or not), as these are not recorded in CPRD data. For example, we could not see whether a clinician considered a patient to be contraindicated for antidepressant treatment, whether antidepressant treatment was declined by the patient, or whether the option of antidepressant treatment was even discussed. Therefore, any interpretation as to why individuals with type 2 diabetes are prescribed less antidepressants than those without, can only be speculative. This is an important area for future research, including qualitive research on clinician and patient attitudes to antidepressant treatment in people with type 2 diabetes.

Antidepressant prescribing is only recommended for people with moderate to severe depression (5). We were unable to adjust for depression severity, as this is not routinely recorded in CPRD data. This could mean that more severe depression in individuals without type 2 diabetes was driving the higher prevalence of antidepressant prescribing. Conceptually, it could be that people with type 2 diabetes were more likely to have mild depression recognized in primary care as they are already in contact with

primary care through their type 2 diabetes; while people without type 2 diabetes might not access primary care unless they have more severe depression. However, we propose that this explanation is unlikely when there is evidence that people with type 2 diabetes in the UK often feel that their mental health is not acknowledged by clinicians (20) and when type 2 diabetes itself is associated with more severe depression (21).

Socioeconomic characteristics are also not recorded in CPRD data. Although we matched participants based on GP practice, which would have accounted for socioeconomic characteristics to an extent, there could still be a considerable amount of unmeasured confounding from socioeconomic status. Socioeconomic deprivation is commonly associated with both antidepressant prescribing (22) and type 2 diabetes (23). However, we would expect to see higher rates of antidepressant prescribing in people with type 2 diabetes, if socioeconomic status was causing considerable confounding. As such, the effect that we observed may have been underestimated.

Explanation of findings and comparison to existing literature

There are a number of potential explanations as to why people with comorbid depression and type 2 diabetes, might be less likely to be prescribed antidepressants than people without type 2 diabetes. There are common side effects of antidepressants that may be particularly problematic for people with type 2 diabetes (11, 13). Antidepressants are known to cause weight gain, which could exacerbate type 2 diabetes. Antidepressants are also known to cause cardiac symptoms, which may be of concern to people with a condition such as type 2 diabetes, which can lead to cardiovascular disease. Although antidepressants were shown to improve glycaemic control in a systematic review of RCTs, they have also been shown in observational studies to be associated with hyperglycaemia (24) and starting insulin (25), as well as hypoglycaemia (26) (though all these may have been subject to confounding by indication). Despite the need to successfully treat depression in order effectively manage physical health in type 2 diabetes, without clear evidence on the benefits and harms of antidepressant medication in this patient group, and accompanying treatment guidelines, it is understandable that clinicians may be wary to prescribe. Indeed, there is evidence to suggest that clinicians under-prescribe in people who take multiple other medications, because of concern about the risk of drug interactions (27). This explanation is further supported by our findings that older people with type 2 diabetes were even less likely to be prescribed antidepressants. Older individuals are more likely to both be prescribed multiple medications and experience medication side effects (28).

Conversely, people with type 2 diabetes are reported to experience a lack of acknowledgement of mental health concerns in clinical care (20). While depression may be recorded on a patient's record, they might not have time to discuss antidepressant treatment or it might not be considered a priority for care. In addition, depression may be confused with diabetes-related distress (29), which is not recommended to

be treated with antidepressant medication (30). Alternatively, they could be more likely to receive psychological therapy instead of antidepressant treatment. However, this information is not available in primary care data. As a result of these, the likelihood of a clinician prescribing of antidepressants to people with type 2 diabetes who have depressive symptoms may be decreased. Our study found that the disparity in antidepressant prescribing in people with type 2 diabetes, was greatest in those who were male and from ethnic minorities. In the general population, both males and people from minoritised ethnic groups have been found to be less likely to receive prescriptions during their medical visits and to use antidepressants (12)(13). Stigma about mental health could make people less likely to accept antidepressant medication (31). This stigma is greater for males (32), older adults (33) and people from minoritised ethnic backgrounds (34). Our study has shown that these inequalities are further increased, when the individual also has type 2 diabetes.

Implications for research and practice

Our findings show that existing demographic inequalities related to antidepressant are exacerbated by having comorbid type 2 diabetes. Mental health needs to be prioritised in people with type 2 diabetes, to ensure that equal access to antidepressants is provided, when appropriate. Further research needs to explore the reasons for this considerable disparity in prescribing. Further research is also urgently required to clarify the benefits and harms of antidepressant prescribing in this patient group. This includes understanding for whom and when antidepressants might be appropriate or inappropriate.

Table 1. Main baseline characteristics of the study population

	Total sample	Type 2 diabetes	Comparison without type 2 diabetes
**Total**	113,812	23,171	90,641
**Sex**			
Male	58,688 (51.6%)	11,998 (51.8)	46,690 (51.2)
Female	55,124 (48.4%)	11,173 (48.2)	43,951 (48.5)
**Age**			
Median age (Q1–Q3)	56<br>(46–66 y)	-	-
> 55 y	57,951 (50.9%)	12,143 (52.4)	45,808 (50.5)
< 56 y	55,861 (49.1%)	11,028 (47.6)	44,833 (49.5)
**Ethnicity**			
White	60,391 (53.1%)	11,203 (48.3)	49,188 (54.3)
Asian	6,248 (5.5%)	2,008 (8.7)	4,240 (4.6)
Black	2,539 (2.2%)	775 (3.3)	1,764 (1.9)
Mixed ethnicity	615 (0.5%)	132 (0.6)	483 (0.5)
Others	639 (0.6%)	160 (0.7)	479 (0.5)
Missing	43,380 (38.1%)	8,893 (38.4)	34,487 (38.0)

Table 2. Results for the association between type 2 diabetes and antidepressant prescribing

	Univariable analysis<br>Odds ratio (95% CI)	Multivariable analysis<br>Odds ratio (95% CI)
**Comparison group without type 2 diabetes**	Reference	
**Exposed group with type 2 diabetes**	0.26 (0.25 to 0.27)	0.25 (0.25 to 0.26)

Table 3. Subgroup analyses stratified by sex, age, and ethnicity

	Exposed group with type 2 diabetes<br>Odds ratio (95% CI)	Comparison group without type 2 diabetes<br>Odds ratio (95% CI)
**Sex**		
Male	Reference	0.23 (0.22 to 0.24)
Female		0.28 (0.27 to 0.29)
**Age**		
Over 55 years old	Reference	0.23 (0.22 to 0.24)
Less than 56 years old		0.28 (0.27 to 0.29)
**Ethnicity**		
White	Reference	0.30 (0.29 to 0.32)
Asian		0.14 (0.12 to 0.16)
Black		0.12 (0.09 to 0.14)
Mixed		0.15 (0.09 to 0.24)
Other		0.15 (0.10 to 0.22)
Missing		0.24 (0.23 to 0.25)

ADDITIONAL INFORMATION

Funding: NA

Ethical approval: The authors assert that all procedures contributing to this work comply with the ethical standards of the relevant national and institutional committees on human experimentation and with the Helsinki Declaration of 1975, as revised in 2008. All procedures involving human subjects/patients were approved by the Independent Scientific Advisory Committee of CPRD (protocol no. 21_001648). All data sent to the CPRD is anonymised and therefore consent is not required.

Competing interests: Annie Jeffery and Joseph Hayes report a relationship with Wellcome Trust that includes: funding grants. Annie Jeffery and Joseph Hayes report a relationship with National Institute for Health Research that includes: funding grants. None of the reported relationships had no role in study design, data collection and analysis, decision to publish, or preparation of the manuscript.

Acknowledgements: Designed the study (AJ, YN), curated the data (AJ), analysed the data (YN), interpreted the findings (all authors), drafted the manuscript (YN), edited the manuscript (all authors).

REFERENCES

1. Harris MD. Psychosocial aspects of diabetes with an emphasis on depression. Curr Diab Rep [Internet]. 2003 [cited 2022 Apr 23];3(1):49–55. Available from: https://pubmed.ncbi.nlm.nih.gov/12643146/

2. Lustman PJ, Anderson RJ, Freedland KE, De Groot M, Carney RM, Clouse RE. Depression and poor glycemic control: a meta-analytic review of the literature. Diabetes Care [Internet]. 2000 Jul 1 [cited 2022 Apr 23];23(7):934–42. Available from: https://diabetesjournals.org/care/article/23/7/934/23870/Depression-and-poor-glycemic-control-a-meta

3. de Groot M, Anderson R, Freedland KE, Clouse RE, Lustman PJ. Association of Depression and Diabetes Complications: A Meta-Analysis. Psychosom Med. 2001 Jul;63(4):619–30.

4. Park M, Katon WJ, Wolf FM. Depression and risk of mortality in individuals with diabetes: a meta-analysis and systematic review. Gen Hosp Psychiatry [Internet]. 2013 May [cited 2023 Sep 25];35(3):217–25. Available from: https://pubmed.ncbi.nlm.nih.gov/23415577/

5. Recommendations | Depression in adults: treatment and management | Guidance | NICE [Internet]. [cited 2022 Aug 10]. Available from: https://www.nice.org.uk/guidance/ng222/chapter/Recommendations

6. National Institute for Health and Care Excellence [Internet]. 2009 [cited 2022 Jan 4]. Overview | Depression in adults with a chronic physical health problem: recognition and management | Guidance | NICE. Available from: https://www.nice.org.uk/guidance/cg91

7. Baumeister H, Hutter N, Bengel J. Psychological and pharmacological interventions for depression in patients with coronary artery disease. Cochrane Database of Systematic Reviews [Internet]. 2011 Sep 7 [cited 2019 Oct 4]; Available from: http://doi.wiley.com/10.1002/14651858.CD008012.pub3

8. British National Formulary [Internet]. 2022 [cited 2022 Jan 4]. SERTRALINE | Drug | BNF content published by NICE. Available from: https://bnf.nice.org.uk/drug/sertraline.html

9. Jeffery A, Buckman JEJ, Francis E, Walters K, Wong ICK, Osborn D, et al. A Systematic Review of Long-term Antidepressant Outcomes in Comorbid Depression and Type 2 Diabetes. medRxiv [Internet]. 2022 Apr 16 [cited 2022 May 3];2022.04.11.22273519. Available from: https://www.medrxiv.org/content/10.1101/2022.04.11.22273519v1

10. Pal K, Sharma M, Mukadam NM, Petersen I. Initiation of antidepressant medication in people with type 2 diabetes living in the United Kingdom —A retrospective cohort study . Pharmacoepidemiol Drug Saf. 2022 Aug 10;

11. Jeffery A, Maconick L, Francis E, Walters K, Wong ICK, Osborn D, et al. Prevalence and characteristics of antidepressant prescribing in adults with comorbid depression and type 2 diabetes mellitus: A systematic review and meta-analysis. Health sciences review (Oxford, England) [Internet]. 2021 [cited 2022 May 2];1:100002. Available from: https://pubmed.ncbi.nlm.nih.gov/35028650/

12. Mars B, Heron J, Kessler D, Davies NM, Martin RM, Thomas KH, et al. Influences on antidepressant prescribing trends in the UK: 1995–2011. Soc Psychiatry Psychiatr Epidemiol

[Internet]. 2017 Feb 1 [cited 2023 Sep 25];52(2):193. Available from: /pmc/articles/PMC5329088/

13. Ahmad G, McManus S, Cooper C, Hatch SL, Das-Munshi J. Prevalence of common mental disorders and treatment receipt for people from ethnic minority backgrounds in England: repeated cross-sectional surveys of the general population in 2007 and 2014. The British Journal of Psychiatry [Internet]. 2022 Sep 18 [cited 2023 Sep 25];221(3):520–7. Available from: https://www.cambridge.org/core/journals/the-british-journal-of-psychiatry/article/prevalence-of-common-mental-disorders-and-treatment-receipt-for-people-from-ethnic-minority-backgrounds-in-england-repeated-crosssectional-surveys-of-the-general-population-in-2007-and-2014/B3B182A20E0D798F164AD3766FC43C90

14. Kautzky-Willer A, Leutner M, Harreiter J. Sex differences in type 2 diabetes. Diabetologia [Internet]. 2023 Jun 1 [cited 2024 Feb 26];66(6):986–1002. Available from: https://pubmed.ncbi.nlm.nih.gov/36897358/

15. Pham TM, Carpenter JR, Morris TP, Sharma M, Petersen I. Ethnic Differences in the Prevalence of Type 2 Diabetes Diagnoses in the UK: Cross-Sectional Analysis of the Health Improvement Network Primary Care Database. Clin Epidemiol [Internet]. 2019 [cited 2024 Feb 26];11:1081. Available from: /pmc/articles/PMC6948201/

16. Chowdhury SR, Chandra Das D, Sunna TC, Beyene J, Hossain A. Global and regional prevalence of multimorbidity in the adult population in community settings: a systematic review and meta-analysis. EClinicalMedicine [Internet]. 2023 Mar 1 [cited 2024 Feb 26];57. Available from: http://www.thelancet.com/article/S2589537023000378/fulltext

17. Clinical Practice Research Datalink | CPRD [Internet]. [cited 2022 Jan 13]. Available from: https://www.cprd.com/

18. Herrett E, Gallagher AM, Bhaskaran K, Forbes H, Mathur R, Staa T van, et al. Data Resource Profile: Clinical Practice Research Datalink (CPRD). Int J Epidemiol [Internet]. 2015 Jun 1 [cited 2022 Jun 15];44(3):827–36. Available from: https://academic.oup.com/ije/article/44/3/827/632531

19. Wolf A, Dedman D, Campbell J, Booth H, Lunn D, Chapman J, et al. Data resource profile: Clinical Practice Research Datalink (CPRD) Aurum. Int J Epidemiol. 2019 Dec 1;48(6):1740-1740G.

20. Chapter 1 - Communication and engagement | Diabetes UK [Internet]. [cited 2024 Feb 26]. Available from: https://www.diabetes.org.uk/for-professionals/improving-care/good-practice/psychological-care/emotional-health-professionals-guide/chapter-1-communication

21. Deleskog A, Ljung R, Forsell Y, Nevriana A, Almas A, Möller J. Severity of depression, anxious distress and the risk of type 2 diabetes – a population-based cohort study in Sweden. BMC Public Health [Internet]. 2019 Aug 27 [cited 2023 Sep 25];19(1). Available from: /pmc/articles/PMC6712830/

22. Von Soest T, Bramness JG, Pedersen W, Wichstrøm L. The relationship between socio-economic status and antidepressant prescription: a longitudinal survey and register study of young adults. Epidemiol Psychiatr Sci [Internet]. 2012 Mar [cited 2022 Aug 31];21(1):87–95. Available from: https://www.cambridge.org/core/journals/epidemiology-and-psychiatric-sciences/article/abs/relationship-between-socioeconomic-status-and-antidepressant-

prescription-a-longitudinal-survey-and-register-study-of-young-adults/98258CD98FAB01C5D068D6AF6FDEE5B0

23. Agardh E, Allebeck P, Hallqvist J, Moradi T, Sidorchuk A. Type 2 diabetes incidence and socio-economic position: a systematic review and meta-analysis. Int J Epidemiol [Internet]. 2011 Jun 1 [cited 2023 Sep 19];40(3):804–18. Available from: https://dx.doi.org/10.1093/ije/dyr029

24. Derijks HJ, Meyboom RHB, Heerdink ER, De Koning FHP, Janknegt R, Lindquist M, et al. The association between antidepressant use and disturbances in glucose homeostasis: Evidence from spontaneous reports. Eur J Clin Pharmacol [Internet]. 2008 May 15 [cited 2022 Mar 14];64(5):531–8. Available from: https://link.springer.com/article/10.1007/s00228-007-0441-y

25. Jeffery A, Bhanu C, Walters K, Wong ICK, Osborn D, Hayes JF. Association between polypharmacy and depression relapse in individuals with comorbid depression and type 2 diabetes: a UK electronic health record study. Br J Psychiatry [Internet]. 2023 Mar 26 [cited 2024 Feb 26];222(3):112–8. Available from: http://www.ncbi.nlm.nih.gov/pubmed/36451601

26. Derijks HJ, Heerdink ER, De Koning FH, Janknegt R, Klungel OH, Egherts AC. The association between antidepressant use and hypoglycaemia in diabetic patients: a nested case-control study. Pharmacoepidemiol Drug Saf [Internet]. 2008 Apr [cited 2022 Mar 14];17(4):336–44. Available from: https://pubmed.ncbi.nlm.nih.gov/18302302/

27. Kuijpers MAJ, Van Marum RJ, Egberts ACG, Jansen PAF. Relationship between polypharmacy and underprescribing. Br J Clin Pharmacol [Internet]. 2008 Jan [cited 2022 Apr 26];65(1):130. Available from: /pmc/articles/PMC2291281/

28. Age UK [Internet]. [cited 2022 Apr 26]. More Harm Than Good. Available from: https://www.ageuk.org.uk/globalassets/age-uk/documents/reports-and-publications/reports-and-briefings/health--wellbeing/medication/190819_more_harm_than_good.pdf

29. Fisher L, Gonzalez JS, Polonsky WH. The confusing tale of depression and distress in patients with diabetes: a call for greater clarity and precision. Diabet Med [Internet]. 2014 Jul 1 [cited 2023 Sep 25];31(7):764. Available from: /pmc/articles/PMC4065190/

30. Diabetes and Mental Health | CDC [Internet]. [cited 2023 Sep 25]. Available from: https://www.cdc.gov/diabetes/managing/mental-health.html

31. Prins MA, Verhaak PFM, Bensing JM, van der Meer K. Health beliefs and perceived need for mental health care of anxiety and depression--the patients' perspective explored. Clin Psychol Rev [Internet]. 2008 Jul [cited 2024 Feb 26];28(6):1038–58. Available from: https://pubmed.ncbi.nlm.nih.gov/18420323/

32. Chatmon BN. Males and Mental Health Stigma. Am J Mens Health [Internet]. 2020 Jul 1 [cited 2024 Feb 26];14(4). Available from: /pmc/articles/PMC7444121/

33. Nair P, Bhanu C, Frost R, Buszewicz M, Walters KR. A Systematic Review of Older Adults' Attitudes Towards Depression and Its Treatment. Gerontologist [Internet]. 2020 Jan 24 [cited 2024 Feb 26];60(1):E93–104. Available from: https://pubmed.ncbi.nlm.nih.gov/31115449/

34. Du N, Chong ESK, Wei D, Liu Z, Mu Z, Deng S, et al. Prevalence, risk, and protective factors of self-stigma for people living with depression: A systematic review and meta-analysis. J Affect Disord [Internet]. 2023 Jul 1 [cited 2024 Feb 26];332:327–40. Available from: https://pubmed.ncbi.nlm.nih.gov/37060952/

Click here to access/download
Supporting Information
PLOSOne_Human_Subjects_Research_Checklist (1).docx
