# Supplementary material for: Antidepressant prescribing inequalities in people with comorbid depression and type 2 diabetes: A UK primary care electronic health record study
Source: PLoS One. 2024 Nov 5;19(11):e0309153. doi: 10.1371/journal.pone.0309153 (PMC11537397; doi:10.1371/journal.pone.0309153)
Supplement: Attachment — Submitted filename: Response to review v2.docx [file pone.0309153.s003.docx]

Thank you for noticing the error in Table 2. We have now updated this with the correct percentages.
